# Supplementary material for: Intracellular Localization and Gene Expression Analysis Provides New Insights on LEA Proteins’ Diversity in Anhydrobiotic Cell Line
Source: Biology (Basel). 2022 Mar 22;11(4):487. doi: 10.3390/biology11040487 (PMC9031878; doi:10.3390/biology11040487)
Supplement: Supplementary file 1 [file biology-11-00487-s001.zip › biology-1599807-supplementaryfinal.pdf]

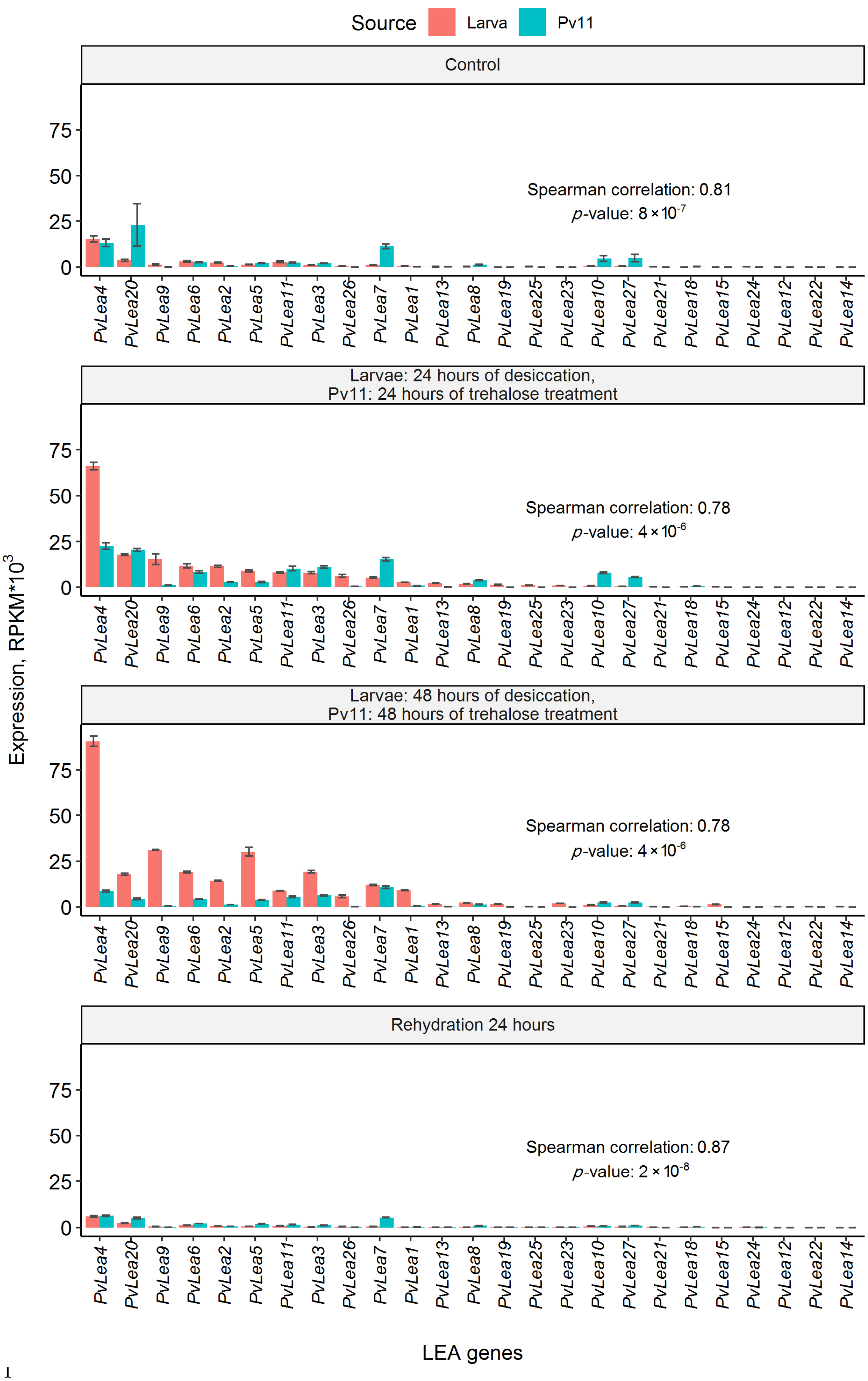

Source
Larva
Pv11
Control
Spearman correlation: 0.81
p-value: 8 × 10^-7
Larvae: 24 hours of desiccation,
Pv11: 24 hours of trehalose treatment
Spearman correlation: 0.78
p-value: 4 × 10^-6
Larvae: 48 hours of desiccation,
Pv11: 48 hours of trehalose treatment
Spearman correlation: 0.78
p-value: 4 × 10^-6
Rehydration 24 hours
Spearman correlation: 0.87
p-value: 2 × 10^-8
Expression, RPKM*10^3
0
25
50
75
PvLea4
PvLea20
PvLea9
PvLea6
PvLea2
PvLea5
PvLea11
PvLea3
PvLea26
PvLea7
PvLea1
PvLea13
PvLea8
PvLea19
PvLea25
PvLea23
PvLea10
PvLea27
PvLea21
PvLea18
PvLea15
PvLea24
PvLea12
PvLea22
PvLea14
LEA genes

1

Figure S1. *PvLea* genes' expression in *P. vanderplanki* larvae and Pv11 cells in control conditions and at different stages of anhydrobiosis induction. The height of the bars depicts mean expression for replicates, whereas error bars show the corresponding standard deviation. The colors of the bars indicate data for larvae (red) or Pv11 cells (blue–green). Genes are ordered in accordance with expression in larvae, and their names are indicated below the plot. Spearman correlation of expression means and the corresponding p–value are indicated by text on the plot. The plot is faceted in accordance with different experimental conditions, as indicated at the top of each panel. In the case of larvae, anhydrobiosis induction is represented by slow desiccation, which takes nearly 48 h up to an air–dry state. In the case of Pv11 cells, which are unable to synthesize trehalose, anhydrobiosis induction consists of trehalose treatment for 48 h, followed by a rapid desiccation (see Methods, section 2.5.).

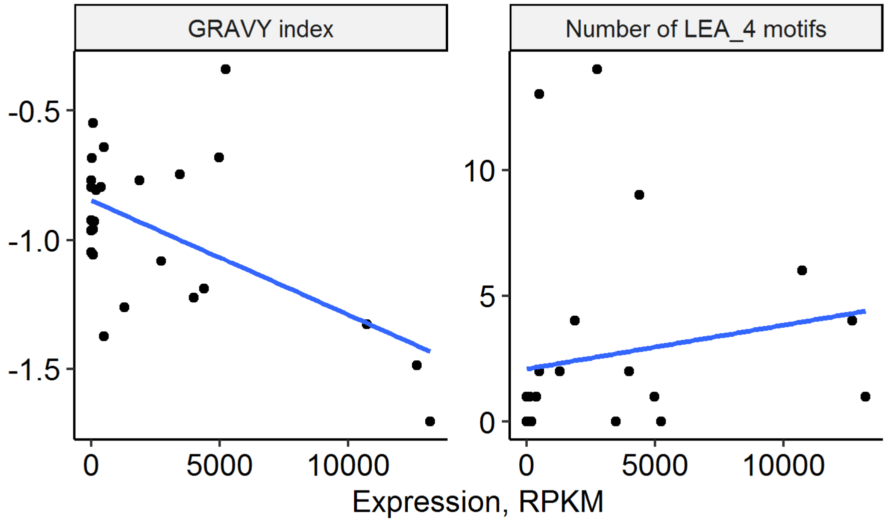

Figure S2. Plot of *PvLea* gene expression in Pv11 cells versus GRAVY index of hydropathy or number of LEA_4 motifs in the respective protein. The GRAVY index is the grand average of hydropathy index, describing protein solubility. Negative GRAVY index values indicate hydrophilic characteristics of the corresponding protein. The GRAVY index data and the number of LEA_4 motifs (ID PF02987 in Pfam 26.0 database) used are from [13]. Mean values of expression for each gene are on the x–axis.

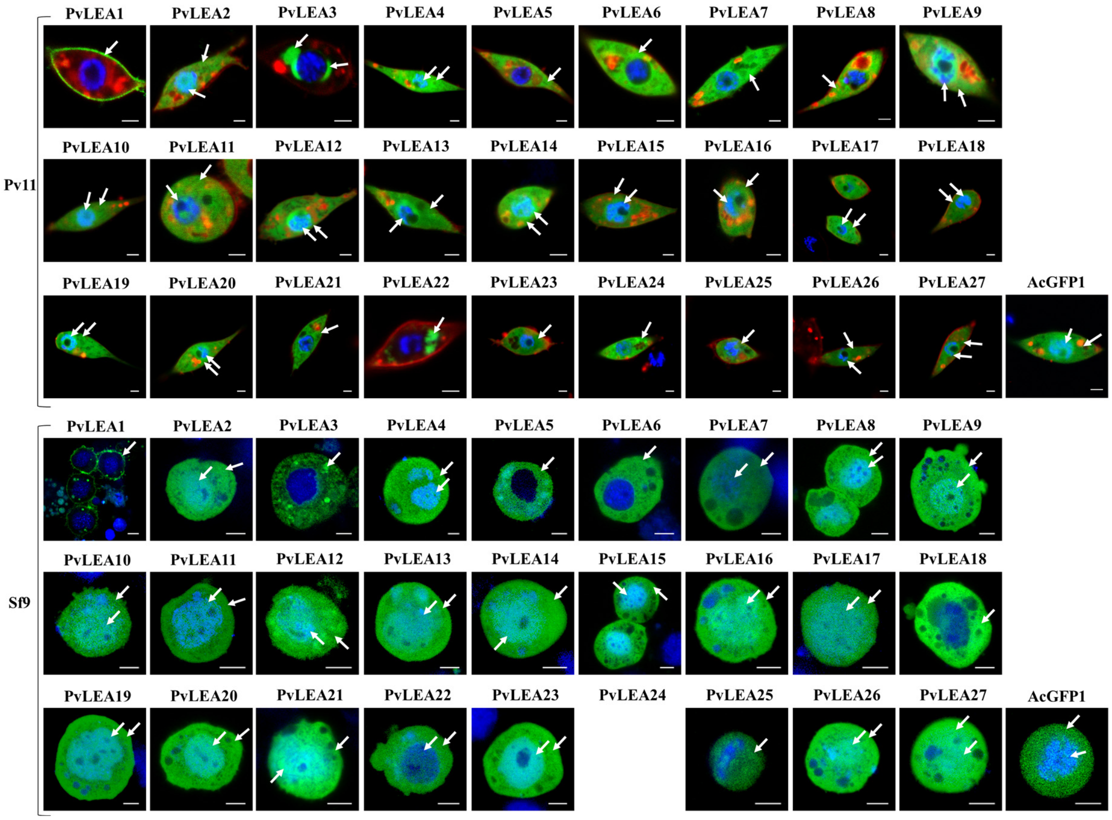

Figure S3. Images of subcellular localization of PvLEA(x)–AcGFP1 chimeras in Pv11 and Sf9 cells (X = 1–27). PvLEA proteins are indicated on the left side of the images, cell cultures and the fusion type at the top. Respective organelles are indicated by arrows. Pv11 cells membranes and DNA in both cell cultures were stained with CellVue Claret Far Red (red) and Hoechst 33258 (blue), respectively. The scale bar is 2 μm for Pv11 cells and 5 μm for Sf9 cells. Green color represents emission of a green fluorescent protein (AcGFP1) expressed in fusion with PvLEA protein. The following filters were used: excitation at 405 nm, emission at 410 – 508 nm (blue channel); excitation at 488 nm, emission at 490 – 633 nm (green channel); excitation at 633 nm, emission at 638 – 759 nm (red channel).

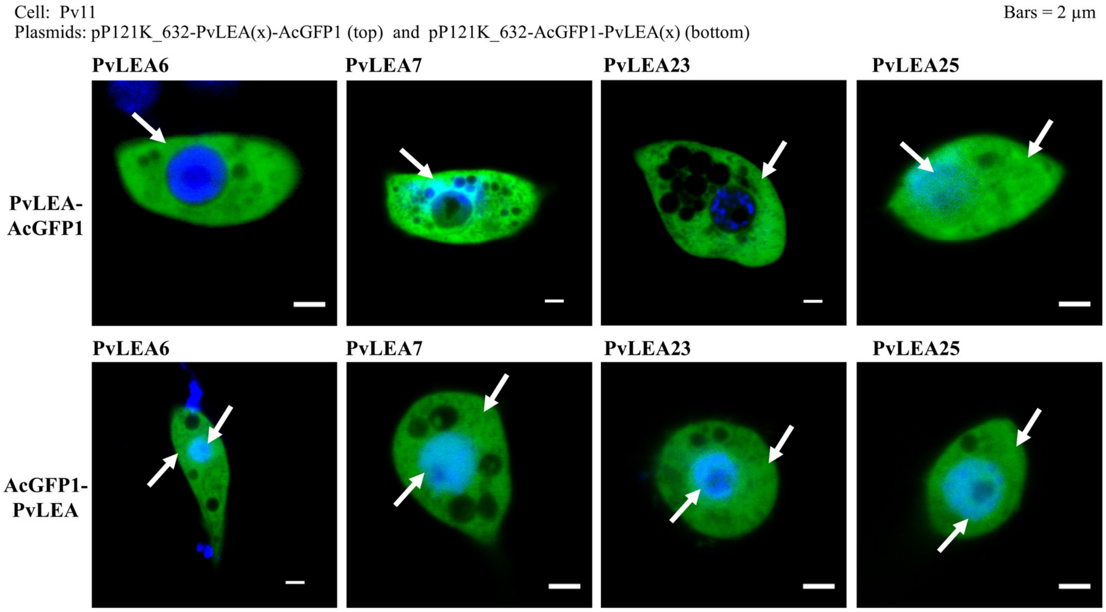

Figure S4. Images of subcellular localization of PvLEA(x)–AcGFP1 and AcGFP1–PvLEA(x) (X = 6, 7, 23 and 25) chimeras under control of the shortened promoter that ensures decreased expression, in comparison to the main set of plasmids (see Methods, section 2.2.). PvLEA proteins are indicated at the top of the images, cell cultures on the left side. Respective organelles are indicated by arrows.

Table S1. Primer sequences (5′–3′).

List of primer sequences.

Primer	Sequence(5'-3')	Note
p121_acgfp_F3	TCCAAGCTTTCTTCTTCTAGATGATATCTAACGCGTACCGGTC ATCATC	Generation of the intermediate vector (pP121K- XhoI-AcGFP-BamHI-HindIII-XbaI-EcoRV)
p121_acgfp_R4	TAGTCTCGAGTTTTTTCAGAAAATATTTTCTTTTGTCCAACG	
acgfp_piz_r2	CCTAAGCTTGGATCCCTTTGTACAGCTCATCCATGCC	
acgfp_piz_f1	TGCCTCGAGCCAAAATGGTGAGCAAGGGCG	
LEA1FW1	AACGGATCCAAATGGTACTAGGAAGTATTTTAA	PvLea1-5 cloning into the intermediate vector (pP121K- XhoI-AcGFP-BamHI-HindIII-XbaI-EcoRV)
LEA1RW1	TGAAGCTCTAGATTAGCAAATTATTTTGTCCATT	
LEA2FW1	AACGGATCCAAATGAAACACGACAAGGGAATTA	
LEA2RW1	TGAAGCTCTAGATTATTTTGGAATTCGTCGCAAAGTG	
LEA3FW1	AACGGATCCAAATGGAGAAAACATTAAACGAG	
LEA3RW1	TGAAGCTCTAGATTACTTAGATTTATCTTCGATTTCGA	
LEA4FW1	AACGGATCCAAATGGTTAAGCAAGATAACT	
LEA4RW1	TGAAGCTCTAGATTAACAACAATTCTTATTCTTATCA	
LEA5FW1	AACGGATCCAAATGAATAACAATCAGTTGGAT	
LEA5RW1	TGAAGCTCTAGATTATTACTTTCTATTCTTGTCGGA	
OpiE2_rev2*	CGCGCTTGAAAGGAGTGTG	Sequencing of AcGFP1-PvLeaX chimers
acgfp_seq_f2	AACCACTACCTGTCCACC	
Scaf121-sf22-F1*	GCATGTCATTACCGGCAATTAC	Sequencing of PvLeaX-AcGFP1-chimers
AcGFP1-R1	GTGGTGCAGATGAACTTCAG	
Lea1_seq_F1	TCCCCTTGACACTTAACTAC	Sequencing of AcGFP1-PvLeaX chimers, additional primers for long PvLea1, PvLea3 and PvLea5 genes
Lea1_seq_F2	TGGACTTGCTGCTGAAAAA	
Lea1_seq_F3	GCAAAGGATGTCACTGCAGA	
Lea3_seq_F1	TATGGCAAGTGAAGGCTATG	
Lea3_seq_R1	ACAATCGCAATTTTGATGCA	
Lea5_seq_F1	CGAAGAAGTCAAAGAAGAAGG	
Lea5_seq_R1	GCAGTGTTGCCTTTTCCATCG	
Lea5_seq_R2	TTTCCATTGTTCCGTCTTTT	

* Scaf121-sf22-F1 and OpiE2_rev2 primers were also used to sequence AcGFP and adjacent cloning sites in the intermediate vector.

Table S2. Correlation of PvLEA protein characteristics with the expression of corresponding genes in Pv11 cells. Rs: values of Spearman correlation. LEA_4 motifs: ID PF02987 in Pfam 26.0 database. GRAVY index: grand average of hydropathy index, describing protein solubility. Negative GRAVY index values indicate hydrophilic characteristics of the corresponding protein. FoldIndex: degree of protein disorder. Data on the number of LEA_4 motifs, FoldIndex and GRAVY index used are from [13]. The p–values were adjusted for multiple hypothesis testing using the Benjamini–Hochberg procedure and for the presence of ties using the permutation procedure (see Methods, section 2.6.).

	By experimental condition			Collectively for all conditions	
**Characteristics**	**Condition**	**Rs**	**p-value**	**Rs**	**p-value**
Number of LEA4 motifs	Control	0.51	0.041	0.48	0
	Anhydrobiosis 24 hours	0.45	0.073		
	Anhydrobiosis 48 hours	0.5	0.041		
	Rehydration 24 hours	0.55	0.033		
GRAVY index	Control	-0.32	0.241	-0.28	0.033
	Anhydrobiosis 24 hours	-0.3	0.243		
	Anhydrobiosis 48 hours	-0.25	0.307		
	Rehydration 24 hours	-0.3	0.243		
Foldindex	Control	-0.18	0.46	-0.12	0.307
	Anhydrobiosis 24 hours	-0.15	0.5		
	Anhydrobiosis 48 hours	-0.09	0.651		
	Rehydration 24 hours	-0.15	0.5		

Table S3. Molecular weight of AcGFP1-PvLEA chimeras, localization consistency of PvLEA fusion proteins and detailed WoLF PSORT predictions of PvLEA localization.

Protein	MW of N-terminal chimera*	Inconsistent localization?	Details of WoLF-PSORT prediction
PvLEA1	110.7		E.R.: 24, extr: 4, pero: 2, plas: 1, golg: 1
PvLEA2	48.1		nucl: 17.5, cyto_nucl: 16.5, cyto: 12.5, mito: 2
PvLEA3	81.9		E.R.: 11, cyto: 9, extr: 4, plas: 3, pero: 3, mito: 2
PvLEA4	43.6		nucl: 19, extr: 8, mito: 3, cyto: 2
PvLEA5	105.8		nucl: 20, cyto: 10, E.R.: 1, cysk: 1
PvLEA6	63.2	+	cyto: 22, nucl: 5, extr: 3, mito: 2
PvLEA7	50.0	+	nucl: 21.5, cyto_nucl: 15, cyto: 7.5, mito: 2, extr: 1
PvLEA8	44.5	+	cyto: 17.5, cyto_nucl: 12, extr: 7, nucl: 5.5, mito: 2
PvLEA9	37.9		cyto: 16.5, cyto_nucl: 11.5, extr: 8, nucl: 5.5, mito: 2
PvLEA10	34.3		nucl: 20, cyto: 12
PvLEA11	40.5		cyto: 17.5, cyto_nucl: 12, extr: 7, nucl: 5.5, mito: 2
PvLEA12	41.2		cyto: 22.5, cyto_nucl: 17.6667, cyto_plas: 13.3333, nucl: 5.5, pero: 2, mito: 1
PvLEA13	39.4		cyto: 21.5, cyto_nucl: 16.5, nucl: 6.5, pero: 2, extr: 1, mito: 1
PvLEA14	37.1		cyto: 21, cyto_nucl: 15, extr: 6, nucl: 5
PvLEA15	36.8		cyto: 18.5, cyto_nucl: 14.5, extr: 6, nucl: 5.5, mito: 1, golg: 1
PvLEA16	36.6		cyto: 22, cyto_nucl: 14.5, extr: 5, nucl: 3, pero: 2
PvLEA17	36.6		mito: 14, cyto: 8.5, cyto_nucl: 8.5, nucl: 7.5, extr: 2
PvLEA18	35.6	+	mito: 19, cyto_nucl: 6, nucl: 5, cyto: 5, extr: 3
PvLEA19	34.9		cyto: 22.5, cyto_nucl: 18.1667, cyto_plas: 13.3333, nucl: 6.5, extr: 2
PvLEA20	33.8		nucl: 24, cyto: 8
PvLEA21	46.5	+	cyto: 23, cyto_nucl: 20, nucl: 7, extr: 2
PvLEA22	46.1	+	cyto_nucl: 19.5, cyto: 17, nucl: 14, mito: 1
PvLEA23	45.6	+	nucl: 18, cyto: 14
PvLEA24	43.8	+	cyto: 19.5, cyto_nucl: 18, nucl: 7.5, extr: 3, pero: 2

PvLEA25	43.5	cyto: 27.5, cyto_nucl: 16.5, nucl: 2.5, extr: 2
PvLEA26	35.4	cyto: 22, cyto_nucl: 13, extr: 8, nucl: 2
PvLEA27	34.9	cyto: 18.5, cyto_nucl: 12.5, extr: 6, nucl: 5.5, mito: 2

Molecular weight of AcGFP1-PvLEA chimeras, localization consistency of fusion proteins and detailed WoLF PSORT predictions of PvLEA localization. Molecular weights of PvLEA N-terminal chimeras with AcGFP1 were computed using Expasy portal (https://web.expasy.org/compute_pi/, accessed 15.01.2022). Localization of a given PvLEA protein was marked as inconsistent if the localization was different for N- and C-terminal fusions with green fluorescent protein (AcGFP1) and/or between cell lines. Color denotes values in respective columns from lowest (white) to highest (bright red); * Deduced molecular weight of PvLEA N-terminal chimeras with AcGFP1. Molecular weight of C-terminal chimeras is 0.3 kDa smaller due to shorter linker sequence.
