# Supplementary material for: Intracellular Localization and Gene Expression Analysis Provides New Insights on LEA Proteins’ Diversity in Anhydrobiotic Cell Line
Source: Biology (Basel). 2022 Mar 22;11(4):487. doi: 10.3390/biology11040487 (PMC9031878; doi:10.3390/biology11040487)

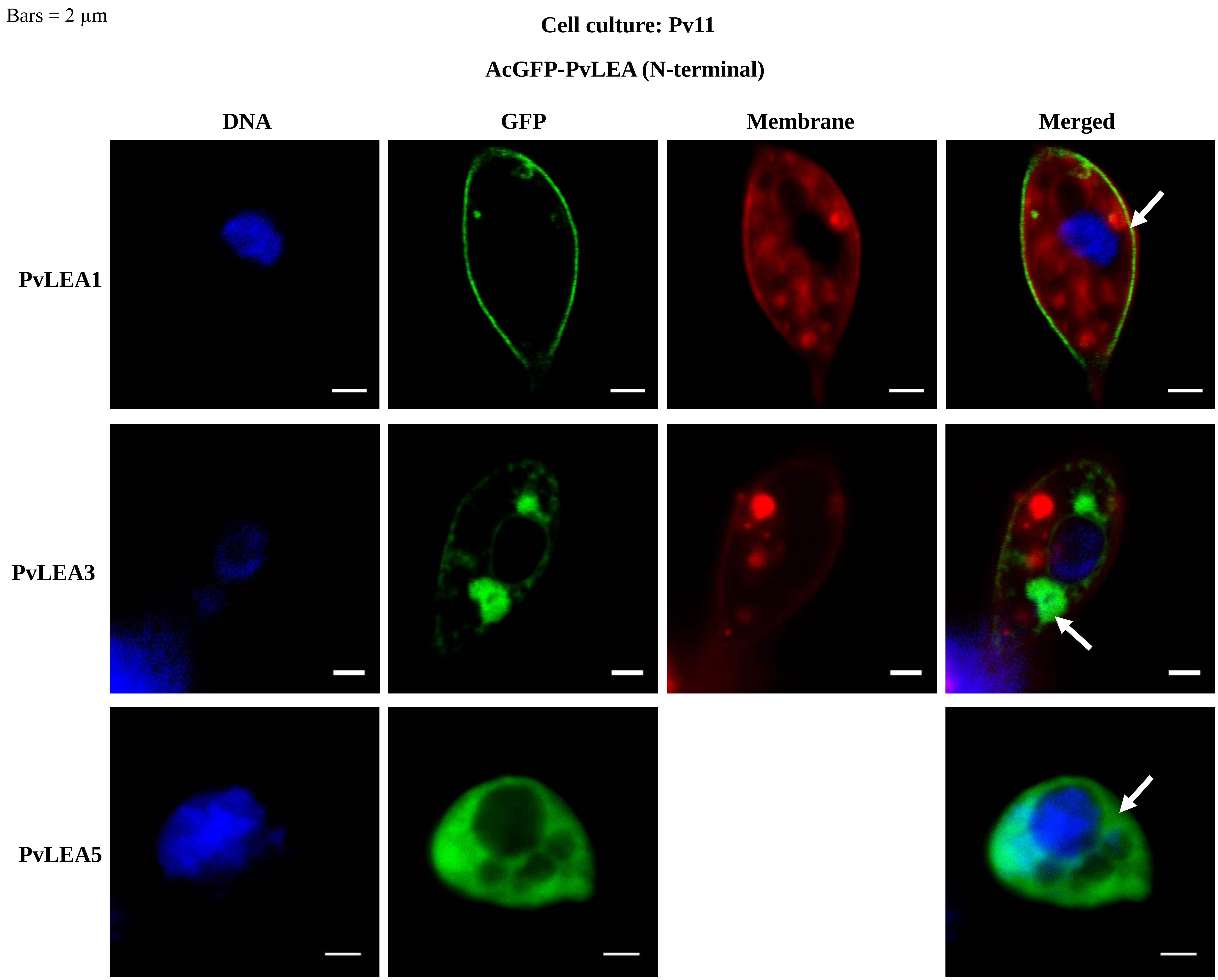
Bars = 2 µm
Cell culture: Pv11
AcGFP-PvLEA (N-terminal)
DNA
GFP
Membrane
Merged
PvLEA1
PvLEA3
PvLEA5

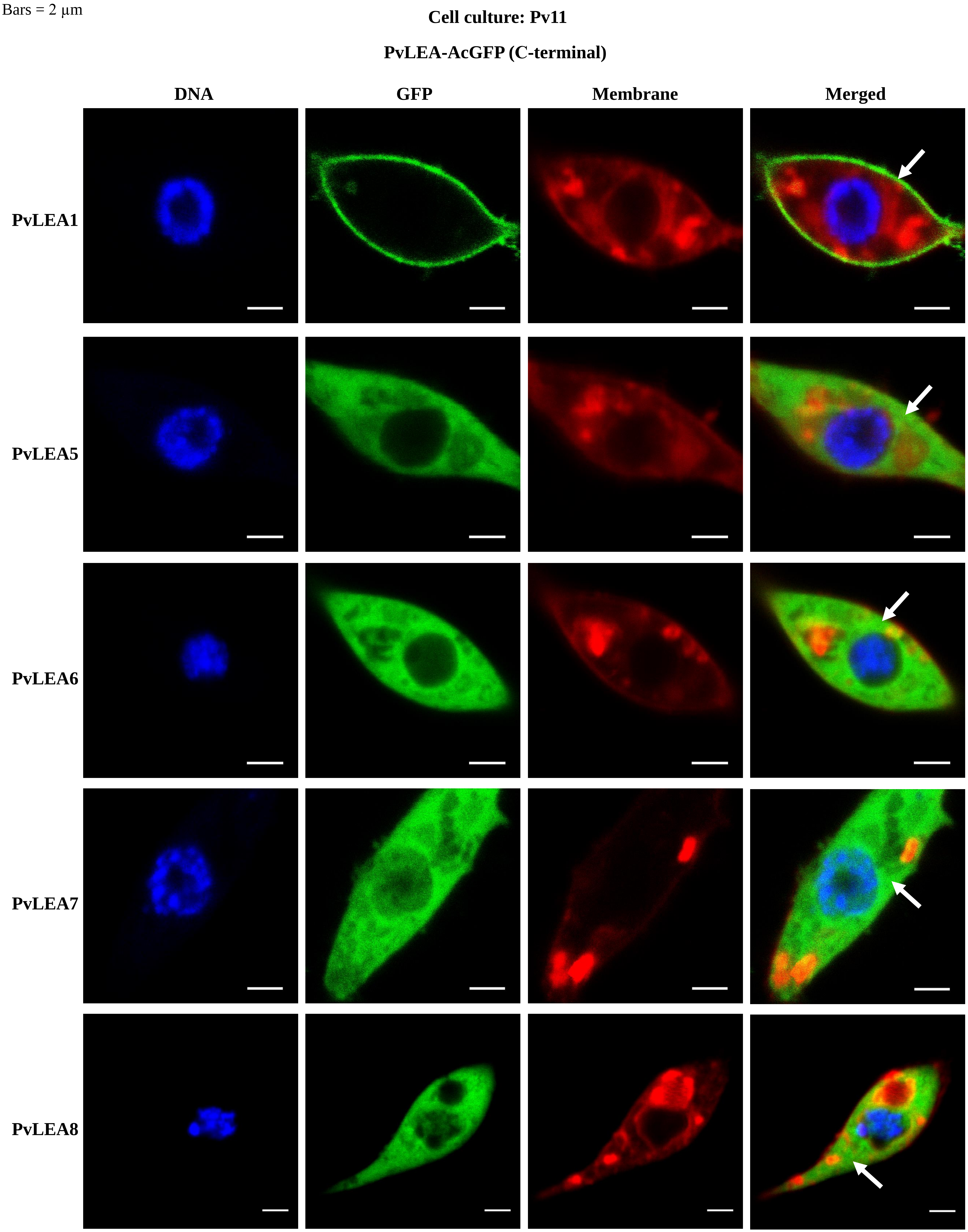
Bars = 2 μm
Cell culture: Pv11
PvLEA-AcGFP (C-terminal)
DNA
GFP
Membrane
Merged
PvLEA1
PvLEA5
PvLEA6
PvLEA7
PvLEA8

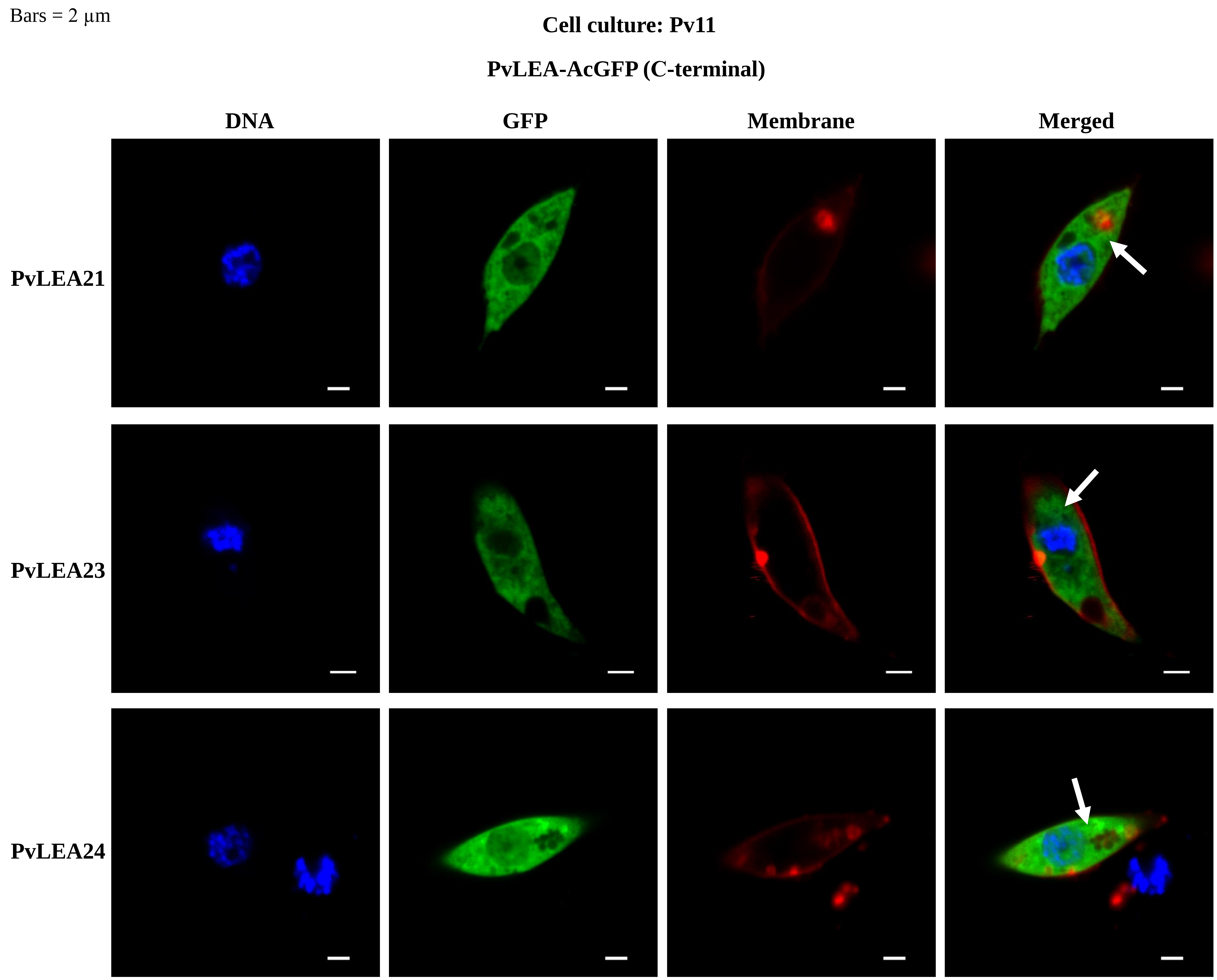

Bars = 2 μm
Cell culture: Pv11
PvLEA-AcGFP (C-terminal)
DNA
GFP
Membrane
Merged
PvLEA21
PvLEA23
PvLEA24

Bars = 5 µm

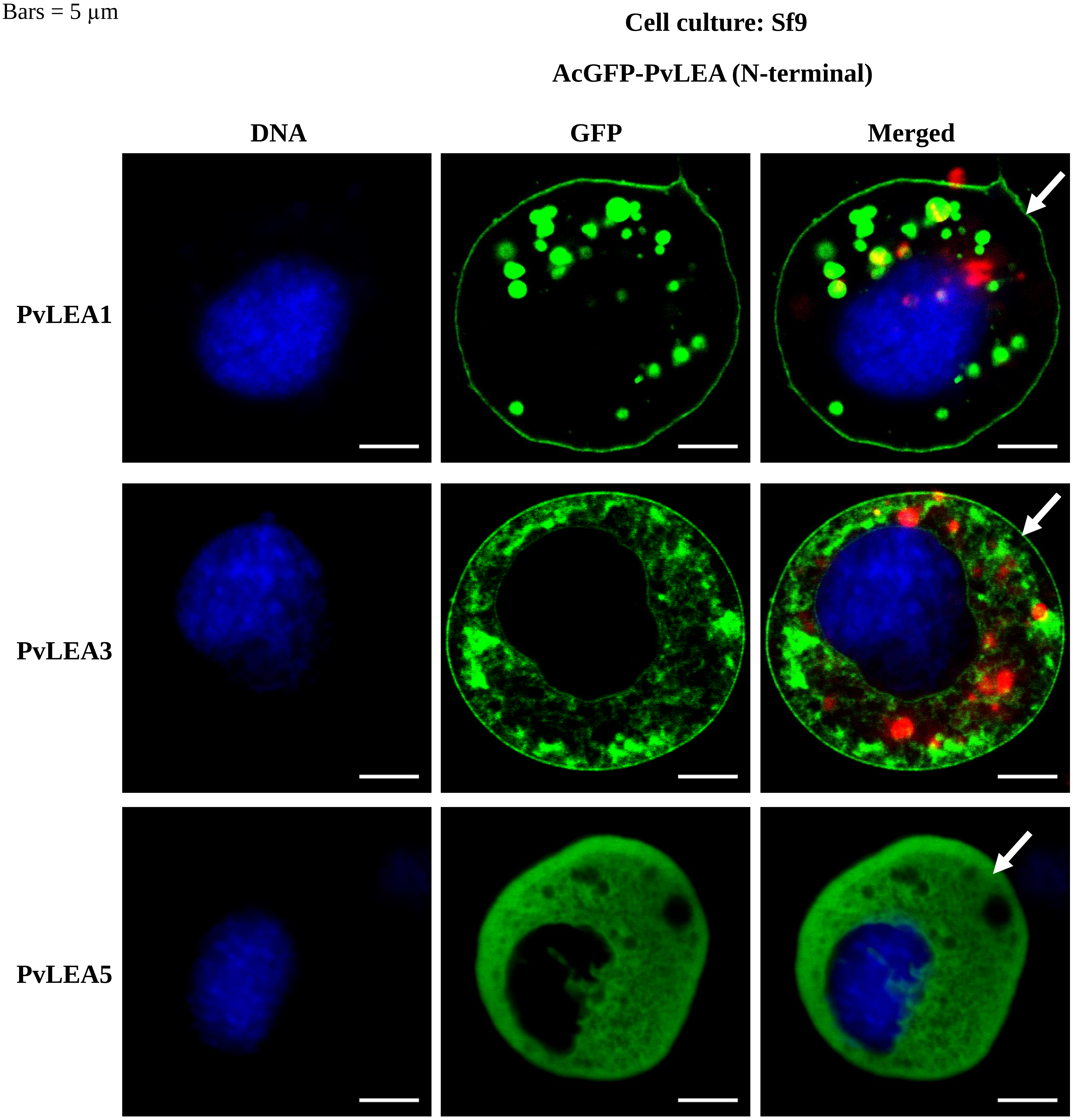
Cell culture: Sf9
AcGFP-PvLEA (N-terminal)
DNA
GFP
Merged
PvLEA1
PvLEA3
PvLEA5

Bars = 5 μm

Cell culture: Sf9

PvLEA-AcGFP (C-terminal)

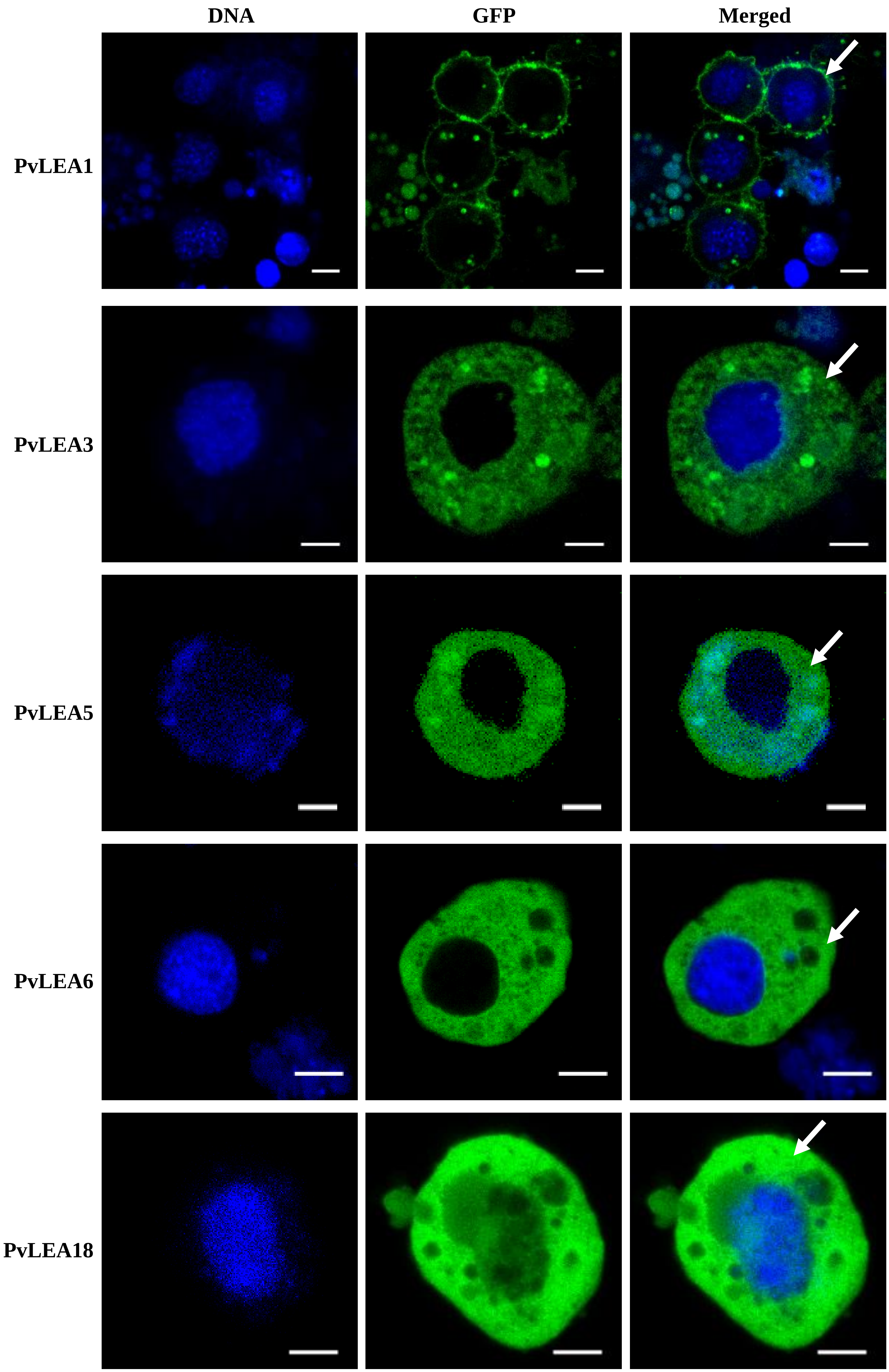

Supplement: Supplementary file 1 [file biology-11-00487-s001.zip › Supplementary Data S1.pdf]
